# The Diet of Diabetic Patients in Spain in 2008–2010: Accordance with the Main Dietary Recommendations—A Cross-Sectional Study

**DOI:** 10.1371/journal.pone.0039454

**Published:** 2012-06-22

**Authors:** Maritza Muñoz-Pareja, Luz M. León-Muñoz, Pilar Guallar-Castillón, Auxiliadora Graciani, Esther López-García, José R. Banegas, Fernando Rodríguez-Artalejo

**Affiliations:** Departamento Medicina Preventiva y Salud Pública, Universidad Autónoma de Madrid/IdiPaz; CIBERESP, Madrid, Spain; Brigham and Women’s Hospital and Harvard Medical School, United States of America

## Abstract

**Background:**

No previous study has assessed the diet of the diabetic patients in the general population of an entire country in Europe. This study evaluates accordance of the diet of diabetic adults in Spain with nutritional recommendations of the European Association for the Study of Diabetes (EASD), American Diabetes Association (ADA), and the Mediterranean diet (MD).

**Methods and Findings:**

Cross-sectional study conducted in 2008–2010 among 12,948 persons representative of the population aged ≥18 years in Spain. Usual food consumption was assessed with a dietary history. EASD accordance was defined as ≥6 points on a score of 12 nutritional goals, ADA accordance as ≥3 points on a score of 6 goals, and MD accordance as ≥7 points on the Mediterranean Diet Adherence Screener. In the 609 diagnosed diabetic individuals, the diet was rich in saturated fat (11.2% of total energy), but trans fat intake was relatively low (1.1% energy) and monounsaturated fat intake was high (16.1% energy). Carbohydrate intake was relatively low (41.1% energy), but sugar intake was high (16.9% energy). Intake of cholesterol (322 mg/day) and sodium (3.1 g/day) was also high, while fiber intake was insufficient (23.8 g/day). EASD accordance was observed in 48.7% diabetic patients, ADA accordance in 46.3%, and MD accordance in 57.4%. The frequency of EASD, ADA and MD accordance was not statistically different between diagnosed and undiagnosed diabetic individuals.

**Conclusions:**

Only about half of diabetic patients in Spain have a diet that is consistent with the major dietary recommendations. The lack of dietary differences between diagnosed and undiagnosed diabetic individuals reflects deficiencies in diabetes management.

## Introduction

Nutritional therapy is an integral component in the prevention and management of diabetes mellitus [1]. In clinical trials, nutritional therapy has shown sustained improvements in glycated hemoglobin (HbA1c) [1]. Moreover, combining this type of therapy with other lifestyle interventions (e.g., physical activity, smoking cessation), can further improve clinical and metabolic outcomes [1]. The Mediterranean diet (MD), in particular, has been shown to reduce the risk of type 2 diabetes [2]. The MD also facilitates weight loss [3], glycemic control, and control of the main cardiovascular risk factors associated with diabetes [4].

Accordingly, various scientific societies have developed recommendations for the nutritional management of diabetes [5]–[8]. Surprisingly, however, only a few population-based studies have examined the diet of persons with diabetes. Two analyses from the National Health and Nutrition Examination Surveys (NHANES) conducted from 1999 to 2002 showed important deficiencies (e.g., excessive intake of saturated fat and low fiber intake) in the diet of a representative sample of adults with diabetes in the US [9], [10]. Low compliance with nutritional recommendations for persons with diabetes has been reported in Europe, but most of these studies were clinic-based [11]–[14] or in participants in cohort studies [15], [16] who are not representative of diabetic persons in the general population. Furthermore, some of these studies recruited only insulin-dependent diabetic patients [11], or elderly men [15]. In addition, two population-based European studies included only local samples [17], [18], and one of them included only elderly persons [18]. Finally, the studies were made at least 10 years ago, so that they may no longer represent the current diet of diabetic subjects.

This article examines the diet of a representative sample of persons with diabetes in Spain in 2008–2010 and assesses the level of accordance with the main dietary recommendations.

## Methods

### Study Design and Participants

The data were taken from the Study on Nutrition and Cardiovascular Risk in Spain (ENRICA), whose methods have been reported elsewhere [19]. This is a cross-sectional study conducted between June 2008 and October 2010 in 12,948 individuals representative of the non-institutionalized Spanish population aged 18 years and over. The sample was first stratified by province and size of municipality of residence. Clusters were then randomly selected in two stages: municipalities and census sections. Finally, within each section households were randomly selected using the directory of fixed telephone lines as the sampling frame. Subjects in the households were selected proportionally to the distribution of the population of Spain by age and sex.

The information was collected in three stages: first, by telephone interview on lifestyles and diagnosed morbidity; second, by household visit to obtain samples of blood and urine samples; and third, by another household visit for anthropometry, measurement of blood pressure and collection of a computerized dietary history. The response rate in the study was 51%.

### Ethics

The study participants provided written informed consent. The ENRICA protocol was approved by the Clinical Research Ethics Committees of the “La Paz” Hospital in Madrid and the “Clinic” Hospital in Barcelona.

### Study Variables

#### Diabetes mellitus

Twelve-hour fasting blood glucose was measured using the oxidase glucose technique (ADVIS 2400 Chemistry System analyzer, Siemens). Diabetes mellitus was defined as glucose ≥126 mg/dl [1] or use of antidiabetic medication. Since nutritional therapy is done only in persons with diagnosed diabetes, the data analyses included only diabetic subjects who responded affirmatively to the question: “Have you ever been told by the doctor that you had diabetes or elevated blood sugar?”.

Glycemic control was assessed by the level of HbA1c, measured by high-performance liquid chromatography (Adams A1c HA-8160, Arkray). Good glycemic control was defined as HbA1c <7% [1].

#### Diet

Diet in the previous year was collected using a computerized dietary history developed from the one used in the EPIC-Spain study [20]–[21]. Nutrient intake was calculated using Spanish food composition tables [19].

**Figure 1:**
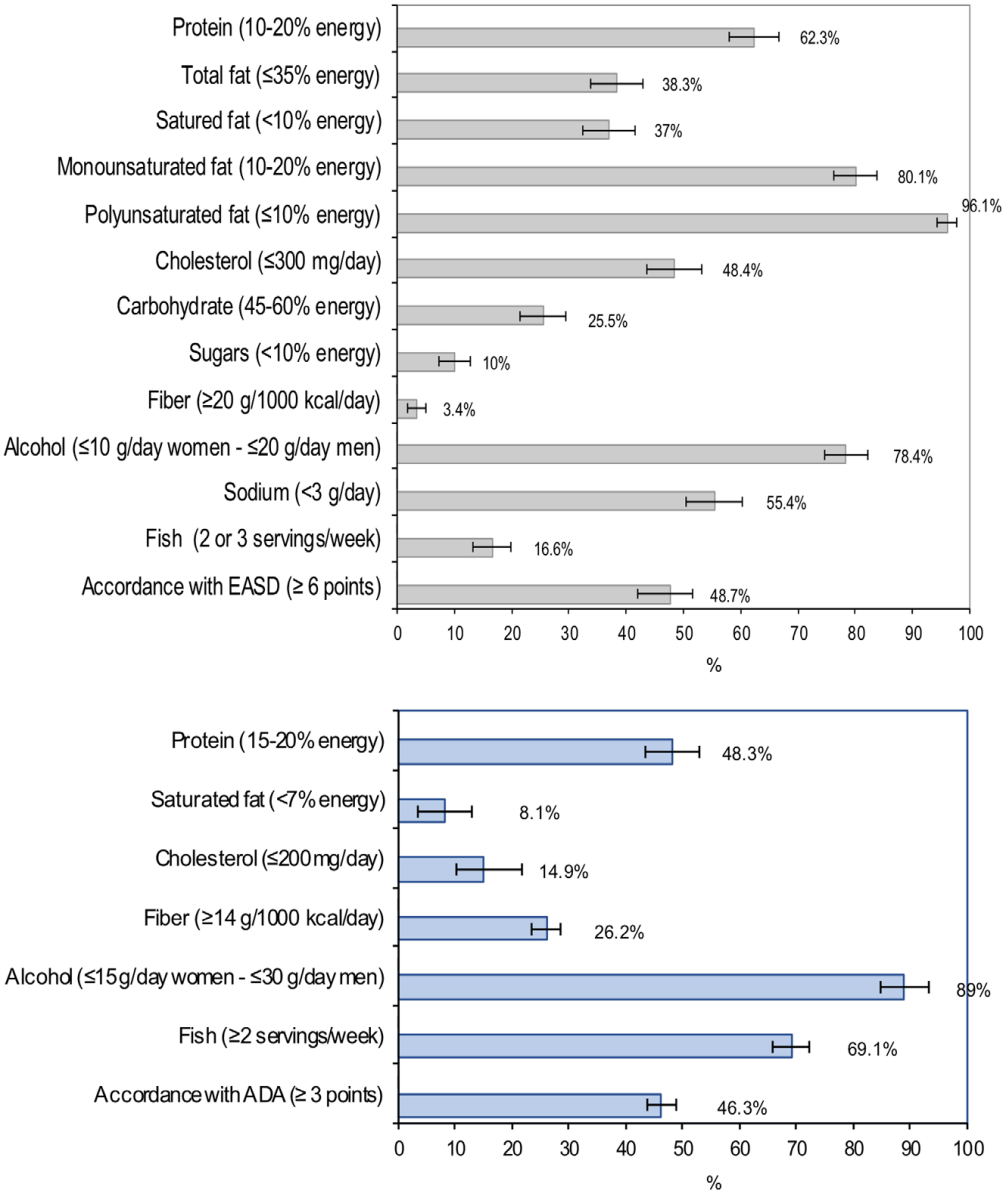
Accordance with nutrient recommendations of EASD and ADA. Percentage of diagnosed diabetic subjects who achieved each nutrient recommendation of the EASD (figure a) and ADA (figure b) and accordance with EASD (figure a) and ADA (figure b). Bars represent 95% confidence intervals.

The quality of the diet of diabetic subjects was evaluated according to the level of accordance with the nutritional recommendations of the European Association for the Study of Diabetes (EASD) [5] and the American Diabetes Association (ADA) [6]. Based on the 12 main nutritional recommendations of the EASD (figure 1), a score was developed that awarded 1 point if the recommendation was met and 0 if it was not met. The final score was calculated as the sum of points for each recommendation (range 0–12); as with similar scores [22], moderate accordance was considered as a score equal to or higher than the intermediate value (≥6 points). An analogous score was constructed to assess accordance with the ADA recommendations. Although the diet recommended by the ADA is consistent with the EASD diet, several ADA recommendations are not formulated quantitatively so that it is not possible to score adherence to them. Consequently, only six recommendations (figure 1) were considered: the ADA score ranged from 0 to 6, and moderate accordance was defined as ≥3 points.

Accordance with the MD was evaluated with the Mediterranean Diet Adherence Screener (MEDAS) [23] developed by the PREDIMED study, a clinical trial of the effect of the traditional MD on the primary prevention of cardiovascular disease [24]. The MEDAS consists of 12 items with goals for food consumption and 2 items with goals for food intake habits characteristic of the Spanish MD. We slightly modified the MEDAS because, in contrast to the original questionnaire, goal achievement for vegetable consumption did not require that at least one of the two daily servings had to be consumed as raw vegetables or a salad. A score of 1 point was assigned for reaching each goal. The MEDAS score ranges from 0 to 14, with a higher score indicating better accordance with the MD. A score of ≥7 was considered as moderate accordance with the MD.

#### Other variables

Study participants reported sociodemographic variables (sex, age, educational level) and lifestyles such as smoking and time spent watching television. Leisure time physical activity was obtained with the EPIC-Spain questionnaire [25], and was expressed in METs-h/week.

Weight, height and waist circumference were measured under standardized conditions [26]. Body mass index (BMI) was calculated as weight in kg divided by height squared in meters, and abdominal obesity was defined as waist circumference >102 cm in men and >88 in women.

Blood pressure was measured with standardized procedures using validated automatic devices (Omron model M6) and three cuff sizes according to arm circumference [27]. Hypertension was defined as systolic blood pressure ≥140 mmHg and/or diastolic blood pressure ≥90 mmHg or drug treatment for hypertension. For the analysis we selected subjects who had been diagnosed with hypertension, that is, hypertensive individuals who responded affirmatively to the question "Have you ever been told by the doctor that you had hypertension, also called high blood pressure?”.

### Statistical Analysis

Of the 12,948 study participants, we excluded the 221 who lacked data on fasting blood glucose. Thus, the analysis was performed with 12,727 individuals, from which we selected the 876 with fasting blood glucose ≥126 mg/dl or who were using antidiabetic medication. From these, we selected the 696 who were aware of their diabetes and, from these, the 635 whose energy intake was within a valid range (for men: >800 to <5000 kcal/day; for women: >500 a <4000 kcal/day) [28], [29]. Finally, we selected those who had complete information for the remaining study variables, resulting in 609 individuals included in the final analytical sample.

The statistical analysis was primarily descriptive: calculation of the percentage and its 95% confidence interval (CI) of persons with diagnosed diabetes whose diet accorded with the recommendations of the EASD, ADA and DM. To summarize the association between the sociodemographic, lifestyle and clinical variables and accordance with the EASD and MD recommendations, we calculated odds ratios (OR) and their 95% CI from logistic models adjusted for sex and age (18–44, 45–64, ≥65 years). To assess the dose-response relation with age, physical activity, time spent watching television and BMI, we modeled the median of each tertile and estimated p-values for linear trend. For educational level, p for trend was based on scores (1, 2, 3) assigned to primary or less, secondary and university education, respectively.

Statistical significance was set as two-sided p<0.05. To take account of the complex sampling design, individual observations were weighted to reconstruct the Spanish population, and the variances were corrected to obtain appropriate 95% CI for the main results. The analyses were performed with the survey procedures of Stata v.11 [30].

## Results

Table 1 shows the main characteristics of subjects diagnosed with diabetes in the ENRICA study. Of note is that over 70% had abdominal obesity and almost 60% suffered hypertension. Two-thirds were being treated only with oral antidiabetic medications. Their diet was rich in saturated fats; however, trans fat intake was relatively low and monounsaturated fat intake was high. Carbohydrate intake was relatively low, but sugar intake was high. Cholesterol and sodium intake were both also high. Finally, only 69.2% of those with diabetes had good glucose control.

**Table 1:** Characteristics of diagnosed diabetic subjects in the ENRICA Study.

	Diagnosed diabeticsn = 609	
**Male sex**		59.1%
**Age,** years		
18–44		7.3%
45–64		36.3%
≥65		56.4%
**Educational level**		
Primary school or less		56.9%
Secondary school		25.2%
University		18.0%
**Smoking**		
Never smokers		48.2%
Past smokers		36.9%
Current smokers		14.8%
**Physical activity,** METs-h/week		23.0 (18.49)
**Hours spent watching TV,** h/week		18.7 (11.23)
**Body mass index,** kg/m^2^		
<25		13.0%
25–29.9		39.9%
≥30		47.1%
**Abdominal obesity**		71.4%
**Hypertension**		57.5%
**Diabetes treatment**		
Oral		67.6%
Insulin		10.1%
Oral+Insulin		9.6%
Without drug treatment		12.6%
**Total energy intake,** kcal/day		2,055 (32.22)
**Dietary intake,**		
Total protein, % of energy		19.2 (3.88)
Total fat, % of energy		36.7 (6.49)
Saturated, % of energy		11.2 (3.14)
*Trans*		1.1 (0.56)
Monounsaturated, % of energy		16.1(3.73)
Polyunsaturated, % of energy		6.1(1.87)
Cholesterol, mg/day		322.2 (129.26)
Carbohydrate, % of energy		41.1(7.14)
Sugars, % of energy		16.9 (5.77)
Fiber, g/day		23.8 (7.99)
Alcohol, g/day		9.1 (15.58)
Total sodium, g/day		3.1 (1.31)
**HbA1c** <7%		69.2%

For continuous variables, the mean (standard deviation) is reported.

Figure 1 shows the percentage of diabetic subjects who followed the EASD and ADA nutritional recommendations. A large percentage met the recommendations for intake of unsaturated fats, alcohol and proteins, but less than 10% reached the goals for sugar and fiber intake, and only 25% met those for carbohydrates. Of note is that although mean fish consumption was high (4 portions/week), the EASD recommends consumption of just 2 or 3 portions/week, therefore only 16.6% had the recommended amount. The mean EASD score was 5.51 (95% CI 5.35–5.69), and accordance with the EASD recommendations (score ≥6 points) was reached in 48.7% of individuals (95% CI 44.0–53.5%).

With regard to the ADA recommendations, only 8% and 15% of individuals, respectively, reached the goals for intake of saturated fats and cholesterol, which are more stringent than those in the EASD recommendations. In contrast, over 25% reached the goal for fiber intake (less stringent than the EASD), and almost 70% reached the goal for fish consumption (≥2 portions/week). The mean ADA score was 2.55 (95% CI 2.45–2.66), and accordance with the ADA recommendations (score ≥3 points) was reached in 46.3% (95% CI 41.7–51.0%) of diabetic subjects. Because compliance with the goals for fiber and fish intake are higher for the ADA than for the EASD recommendations, calculation of the EASD score using the ADA recommendations for fiber and fish consumption increased the percentage of subjects with moderate accordance with EASD, from 48% to 62%.

In the analyses adjusted for age and sex, accordance with EASD recommendations increased with age, and was higher in women and in individuals with hypertension (table 2).

**Table 2:** Variables associated with accordance with the EASD diet.

	Accordance with EASD diet	
	% (95% CI)	Odds ratio (95% CI) a
**Sex**		
Male	41.9 (35.8–47.8)	1.00 (Ref.)
Female	58.8 (51.5–66.1)	1.79 (1.21–2.65)
**Age,** years		
18–44	29.6 (13.8–45.4)	1.00 (Ref.)
45–64	39.9 (32.2–47.6)	1.64 (0.70–3.82)
≥65	56.9 (50.9–62.9)	2.96 (1.30–6.75)
* p for trend*		<0.001
**Educational level**		
Primary school or less	55.1 (48.9–61.2)	1.00 (Ref.)
Secondary school	40.5 (31.2–49.7)	0.75 (0.46–1.23)
University	40.3 (29.8–50.9)	0.76 (0.47–1.27)
* p for trend*		0.219
**Smoking**		
Never smokers	43.7 (32.2–55.1)	1.00 (Ref.)
Past smokers	44.4 (36.3–52.4)	0.96 (0.59–1.56)
Current smokers	53.6 (46.9–60.5)	1.01 (0.57–1.78)
**Physical activity,** METs-h/week		
Tertile 1 (<13.5)	52.1 (43.9–60.4)	1.00 (Ref.)
Tertile 2 (≥13.5 to <26.3)	46.4 (38.4–54.4)	0.86 (0.54–1.40)
Tertile 3 (≥26.3)	47.9 (39.4–56.5)	1.21 (0.74–1.95)
* p for trend*		0.445
**Hours spent watching TV,** h/week		
Tertile 1 (<14)	43.9 (34.9–52.8)	1.00 (Ref.)
Tertile 2 (≥14 to <21)	50.9 (41.6–60.2)	1.25 (0.74–2.11)
Tertile 3 (≥21)	50.9 (44.0–57.8)	1.03 (0.65–1.65)
* p for trend*		0.928
**Body mass index,** kg/m^2^		
<25	51.6 (38.9–64.3)	1.00 (Ref.)
25–29.9	46.5 (38.8–54.3)	0.77 (0.42–1.43)
≥30	49.9 (42.9–56.9)	0.85 (0.45–1.59)
* p for trend*		0.842
**Abdominal obesity**		
No	48.8 (40.3–57.4)	1.00 (Ref.)
Yes	48.7 (43.0–54.5)	0.88 (0.57–1.37)
**Hypertension**		
No	40.4 (33.3–47.6)	1.00 (Ref.)
Yes	54.9 (48.9–60.9)	1.60 (1.09–2.35)
**Diabetes treatment**		
Oral	50.1 (44.3–55.8)	1.00 (Ref.)
Insulin	48.9 (33.6–64.2)	1.23 (0.64–2.34)
Oral+Insulin	50.6 (35.9–65.3)	1.00 (0.52–1.93)
Without drug treatment	40.3 (26.9–53.6)	0.86 (0.46–1.59)

a Odds ratio adjusted for sex and age.

n = 609.

**Figure 2:**
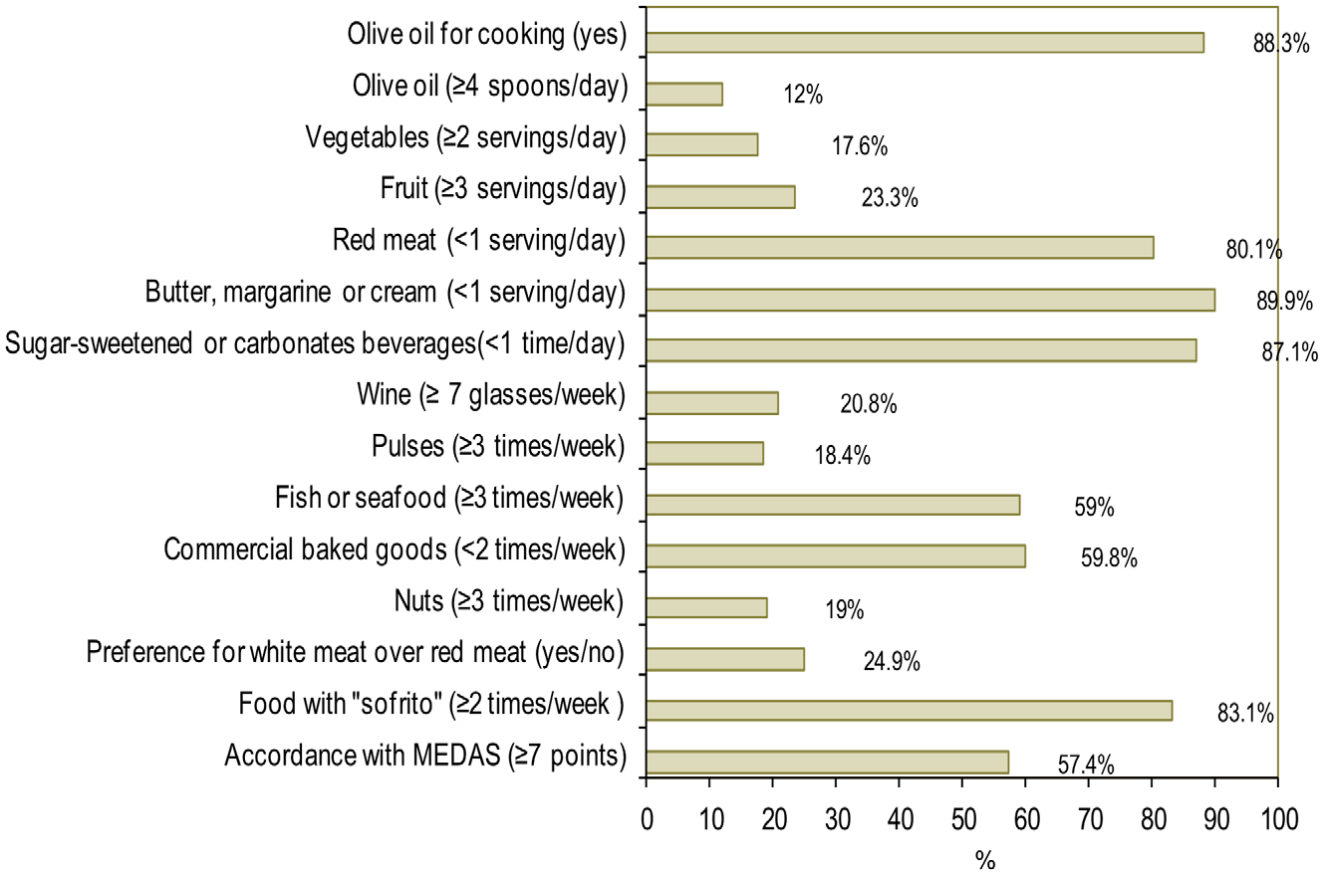
Accordance with the Mediterranean diet. Percentage of diagnosed diabetic subjects who achieved each target of the Mediterranean Diet Adherence Screener (MEDAS) and accordance with the Mediterranean diet. Bars represent 95% confidence intervals. Sofrito: Sauce made with tomato and onion, leek, or garlic and sautéed in olive oil.

Figure 2 presents the percentage of diabetic individuals who followed the MD as assessed with MEDAS. Over 80% reached the goals on use of olive oil as the main cooking oil, consumption of red meat, animal fats, carbonated or sugar-sweetened beverages, and foods prepared with “sofritos” (a commonly used Spanish sauce made with tomato and onion, leek and/or garlic sautéed in olive oil). Furthermore, almost 60% of diabetic subjects consumed ≥3 portions/week of fish, and a similar proportion consumed commercial desserts <2 times/week. In contrast, less than 20% reached the goals for consumption of vegetables, pulses, and nuts. The mean MEDAS score was 6.83 (95% CI 6.65–7.02), and accordance with the MD (≥7 points) was reached in 57.4% (95% CI 52.6–62.3%) of individuals. After excluding the goal on wine consumption, accordance with the MD rose to 74.9% (95% CI 70.3–79.3%) (score ≥6 points on MEDAS with 13 goals). However, only 0.4% of subjects met all 14 dietary goals of MEDAS.

Moderate accordance with the MD was higher in persons ≥65 years of age, but was lower in women (table 3). However, after eliminating the goal on wine consumption from the MEDAS score, the association with sex disappeared (OR 0.79; 95% CI 0.50–1.27).

**Table 3:** Variables associated with accordance with Mediterranean Diet.

	**Accordance with Mediterranean Diet**	
	**% (95% CI)**	**Odds ratio (95% CI)** a
**Sex**		
Male	62.1 (55.6–68.5)	1.00 (Ref.)
Female	50.7 (43.4–58.1)	0.60 (0.41–0.89)
**Age,** years		
18–44	38.8 (20.8–57.2)	1.00 (Ref.)
45–64	58.7 (50.4–67.1)	2.21 (0.95–5.14)
≥65	59.0 (52.9–65.2)	2.46 (1.09–5.54)
* p for trend*		0.070
**Educational level**		
Primary school or less	55.1 (48.8–61.3)	1.00 (Ref.)
Secondary school	59.0 (49.4–68.6)	1.23 (0.76–1.99)
University	62.7 (51.3–74.1)	1.33 (0.77–2.31)
* p for trend*		0.251
**Smoking**		
Never smokers	55.9 (49.2–62.6)	1.00 (Ref.)
Past smokers	58.1 (50.3–65.9)	0.75 (0.47–1.20)
Current smokers	60.9 (49.5–72.4)	1.00 (0.55–1.80)
**Physical activity,** METs-h/week		
Tertile 1 (<13.5)	51.1 (43.0–59.1)	1.00 (Ref.)
Tertile 2 (≥13.5 to <26.3)	60.7 (52.6–68.7)	1.42 (0.89–2.25)
Tertile 3 (≥26.3)	60.3 (51.5–69.1)	1.43 (0.86–2.36)
* p for trend*		0.158
**Hours spent watching TV,** h/week		
Tertile 1 (<14)	55.1 (45.7–66.5)	1.00 (Ref.)
Tertile 2 (≥14 to <21)	63.9 (54.9–73.1)	1.45 (0.86–2.47)
Tertile 3 (≥21)	55.3 (48.3–62.3)	0.98 (0.60–1.59)
* p for trend*		0.842
**Body mass index,** kg/m^2^		
<25	53.8 (41.2–66.4)	1.00 (Ref.)
25–29.9	59.3 (51.6–67.0)	1.12 (0.61–2.05)
≥30	56.9 (50.0–63.7)	1.06 (0.59–1.93)
* p for trend*		0.946
**Abdominal obesity**		
No	63.9 (55.8–72.0)	1.00 (Ref.)
Yes	55.1 (49.3–60.9)	0.73 (0.48–1.11)
**Hypertension**		
No	54.1 (46.6–61.6)	1.00 (Ref.)
Yes	59.9 (53.8–66.1)	1.23 (0.83–1.82)
**Diabetes treatment**		
Oral	58.1 (56.2–64.0)	1.00 (Ref.)
Insulin	56.5 (40.7–72.3)	1.20 (0.60–2.43)
Oral+Insulin	55.8 (41.1–70.6)	0.93 (0.48–1.81)
Without drug treatment	55.9 (41.8–70.2)	0.95 (0.52–1.76)

a Odds ratio adjusted for sex and age.

n = 609.

Individuals with MD accordance more frequently had good glucose control (HbA1c <7%), both in the analysis adjusted for age and sex and in the one additionally adjusted for various lifestyle and clinical variables (table 4). Moreover, in the linear regression analysis, the higher the MEDAS score, the lower the HbA1c level (fully-adjusted β −0.055%/1-unit increase in MEDAS score; 95% CI −0.11 to −0.004%/1-unit in MEDAS score). The results for accordance with the EASD and ADA recommendations were in the same direction, but did not reach statistical significance (table 4).

**Table 4:** Association of accordance with EASD diet, ADA diet and Mediterranean Diet with glycemic control.

	HbA1c <7%		
	% (95% CI)	Odds ratio (95% CI)^a^	Odds ratio (95% CI)^b^
**Accordance with EASD**			
No	49.9 (44.2–55.6)	1.00 (Ref.)	1.00 (Ref.)
Yes	50.1 (44.4–55.8)	1.13 (0.73–1.74)	1.06 (0.66–1.70)
**Accordance with ADA**			
No	50.9 (45.5–56.2)	1.00 (Ref.)	1.00 (Ref.)
Yes	49.1 (43.8–54.5)	1.37 (0.91–2.10)	1.34 (0.87–2.07)
**Accordance with MD**			
No	38.6 (32.9–42.3)	1.00 (Ref.)	1.00 (Ref.)
Yes	61.4 (55.7–67.1)	1.61 (1.05–2.47)	1.56 (1.1–2.45)

a Model adjusted for sex and age.

b Model adjusted for sex, age, educational level, smoking, physical activity, hours spent watching TV, body mass index, abdominal obesity, hypertension and diabetes treatment.

n = 609.

Finally, we compared the diet of the 609 diagnosed diabetic persons with the 180 who were not aware of their diabetes in the ENRICA study. In the analyses adjusted for age and sex, there were no statistically significant differences between these two groups in the frequency of accordance with the recommendations of the EASD (OR 0.95; 95% CI 0.63–1.94), ADA (OR 1.16; 95% CI 0.76–1.76) or MD (OR 0.86; 95% CI 0.56–1.33). Moreover we found no evidence that differences in healthy diet accordance by diagnostic status varied with age (p for interaction >0.20 in all cases).

## Discussion

### Interpretation of the Key Findings

Our results show that only about half of Spaniards with diabetes have a diet accordant with the recommendations of the EASD, ADA or the MD pattern. Moreover, the absence of dietary differences between diagnosed and undiagnosed individuals with diabetes suggests deficiencies in the health care system in the management of diabetes.

Although there is room for improvement, the diet of persons with diabetes in Spain has some healthy characteristics that would be expected in Mediterranean countries. Specifically, the high intake of mono- and poly-unsaturated fat, which is consistent with the high consumption of olive oil, fish and plant-based foods. Furthermore, although achievement of the goals for consumption of fiber, fruits and vegetables is low (probably because the goals are quite stringent), consumption of these products was fairly substantial. Mean fiber intake was 23.8 g/day, and mean intake of fruits and vegetables was 305 g/day (2.5 servings) and 277 g/day (1.4 servings), respectively.

Thus, the diet of diabetic persons in Spain is consistent with the so-called “evolved Mediterranean diet” (EMD), which is the diet of the Spanish population today [31]–[33]. The EMD maintains considerable intake of olive oil and plant-based foods and moderate consumption of fish, which are typical of the MD, but has incorporated excessive consumption of foods characteristic of the Western diet in industrialized countries, such as animal products rich in saturated fats and cholesterol, and foods high in sugar, which displace complex carbohydrates. Nonetheless, carbohydrates and monounsaturated fat still represent 57.2% of total energy in diagnosed diabetic persons. Salt consumption in the EMD is also excessive, so that only half of diabetic individuals consume <3 g sodium/day. This is important because more than half of those diagnosed with diabetes have also been diagnosed with hypertension.

Among diabetic subjects, accordance with the main food recommendations increased with age. This is consistent with the results of studies in the general population in Spain, which have found that older adults have higher compliance with nutritional goals and higher adherence to the MD [34], [35]. This is likely due to cultural reasons, and to the fact that it is easier for older people to cook and eat at home.

Comparison of our results with those of previous studies is difficult due to methodological differences in the participant’s selection (e.g. clinic-based, cohort-based or population-based sampling) and in instruments used to collect dietary data (food frequency questionnaire, 12-hour recall, 7-day records, diet history). Nevertheless, the literature shows that the diet of diabetic subjects is similar to that of the general population in each country [15], [17], [18] or that of non-diabetic persons in the same cohort [16]. As in our study, Rivellese et al reported that people with type II diabetes in Italy had adequate consumption of mono- and poly-unsaturated fats, but excess consumption of saturated fats and low fiber intake [12]. In another study conducted in six Mediterranean countries, Thanapoulou et al reported low consumption of carbohydrates and fiber among diabetic participants [17].

In comparison with the ENRICA study, diabetic subjects aged 51–84 in the 1999–2000 NHANES showed higher ADA-based compliance with the goals for intake of protein (70%) and cholesterol (49%) [9]. Likewise, diabetic persons in the 1999–2002 NHANES were seen to have higher compliance with intake of proteins (64%) and saturated fats (48%) [10]. In contrast, in both studies fiber intake was lower than in the ENRICA study. This may be due to greater focus on controlling saturated fat and cholesterol intake in the US diet than in Spain, and to higher fruit and vegetable consumption in Spain than in the US [31], [33], [36].

Our results suggest that diet quality in persons with diabetes in Spain has improved in the last decade. In a study conducted in the year 2000 in four outpatient diabetes clinics [13], less than 10% of diabetic patients consumed <10% of energy in the form of saturated fat and <8% ingested <300 mg/day of cholesterol, versus 37% and 48%, respectively, in the ENRICA study. Moreover, fiber intake was lower than in ENRICA.

As expected, higher accordance with dietary recommendations and, in particular, with the MD was associated with better glycemic control. This suggests that clinical trial evidence on the efficacy of MD can be translated into effective interventions within routine clinical practice with unselected diabetic populations [4]. Other cross-sectional analyses with clinic-based samples have also observed an association between the MD and glycemic control in diabetic subjects [37], [38].

Finally, we should note the lack of differences between persons with diagnosed and undiagnosed diabetes in the level of accordance with the EASD and MD recommendations. This suggests problems in the implementation of nutritional guidelines, in education in diabetes management, and in self-care of diabetic patients in Spain. Future investigations should examine the barriers in clinical practice to nutritional education and the determinants of proper nutrition in diabetic individuals.

### Limitations

This study has certain strengths and methodological limitations. Among its strengths are that it was conducted in a representative sample of an entire country, glucose was measured in a central laboratory, and a validated instrument was used to measure the diet.

One of its limitations is the cross-sectional design, which does not allow to infer causality for some of the associations found (e.g., lower HbA1C with increasing adherence to the MD). The cross-sectional design or insufficient statistical power may also have impeded observation of associations that would be expected a priori, such as the association between adherence to EASD or MD recommendations and less obesity. The response rate (51%) in the ENRICA study is also a cause of concern, although it should be noted that it is among the highest of the National Health Interview and Examination Surveys conducted in Europe [39]. However, we cannot rule out that those who were more health-conscious were more inclined to participate. As a result, the accordance with nutritional guidelines among diabetic adults observed in this study could even be higher than the actual one. Although observed sodium intake was excessive, it is also likely to be an underestimate because self-reported intakes are usually lower that those derived from 24-h urinary sodium excretion [40]. Another limitation is that the definition of diabetes was based on a single blood glucose measure, and the diagnosis of diabetes was self-reported, which means that classification error cannot be excluded. Finally, there are several scales to measure the MD, and the results may vary according to the scale used. However, the MEDAS was specifically developed and validated in a Spanish population [23].

### Conclusions

Our study shows an important gap between the nutritional recommendations of scientific societies and the diet in diabetic individuals in Spain. Moreover, the lack of dietary differences between diagnosed and undiagnosed persons points to deficiencies in the health care system, both in nutritional education for diabetic patients and in support for self-care. Finally, diabetic patients in Spain should reduce their intake of saturated fats, cholesterol, sugar and salt, and should increase their intake of complex carbohydrates and fiber.
